# A Report of the Facts and Circumstances Relating to a Case of Compound Fracture, and Prosecution for Malpractice, in Which William Smith Was Plaintiff, and Drs. Goodyear and Hyde Were Defendants, at Cortland Village, Cortland County, N. Y., March, 1841, Comprising Statements of the Case by Several Medical Gentlemen, Together with Notes and Comments on the Testimony

**Published:** 1841-11-06

**Authors:** 


*A Report of the Facts and Circumstances relating to a case of Compound Fracture, and Prosecution for Malpractice, in which William Smith was Plaintiff, and Drs. Goodyear and Hyde were Defendants, at Cortland village, Cortland county, N. Y., March,* 1841, *comprising Statements of the case by several Medical Gentlemen, together with Notes and Comments on the Testimony.* By A. B. SHIPMAN, M. D., Cortlandville, 1841. pp. 35.

It is certainly the least pleasant of all the duties of a medical reviewer to notice those professional disputes which are submitted to the arbitrament of law. For, independent of the painfulness of witnessing such discussions and reflecting upon their reaction on the public reputation of the fraternity, the bungling and imperfect manner in which the reports of evidence in criminal cases in this country are registered, renders them almost incomprehensible both to the medical and general reader.

Every one is aware that in a rapid examination—especially when a witness is terse and decided in his style of speech—the question itself essentially modifies the meaning of the reply; and, by a most wholesome custom prevalent in England, both are deliberately recorded. In this country, on the contrary, the question is usually neglected, and if the answer is so worded as to convey no meaning without reference to the inquiry, its phraseology is often altered at the discretion of the reporter—a discretion upon which exceedingly little reliance can be placed in medico-legal cases. Sometimes, when the reply is inconveniently long, it is ingeniously curtailed.

Let us give an example from the celebrated trial of Mina, the Mexican, for the murder of Mr. Chapman. We were then giving evidence on the diagnostic appearances after death, in common and spasmodic cholera, and in poisoning by arsenic. The quotation is from memory, but is substantially correct.

Question, (in death from common cholera)—"What were the appearances in the abdomen?"

Answer.—"I found *the veins conveying the blood from* the stomach and bowels *to the liver*, full and distended."

The words written in Italics being ommitted by the reporter, the printed account of the trial read thus:

"Found the stomach and bowels full and distended!"

Yet such are the records which, if not admitted as evidence of the highest character in future trials, are often employed by council to influence the minds of juries in cases involving the lives of our fellow citizens; and such are the statements upon which the medical witness is too frequently judged by his professional brethren and the general public. We have long ceased to consider the ordinary publications of American medico-legal trials as safe foundations for judgments upon the professional character of witnesses, unless where the evidence is so purely narrative as to leave no reasonable room for doubt. But in the pamphlet before us there are many circumstances in evidence of a nature scarcely to be mistaken, and the most important of these are supported by much concurrent testimony, as well as other documentary proof.

It appears that Dr. Shipman has been rudely attacked in a public newspaper, in Cortland county, N. Y., for endeavouring to save a limb in a case of compound fracture of the leg, which was condemned to amputation by two of his brother practitioners, and feels compelled to appeal to the public in self-defence. The facts of the case, as presented, are simply these:

W. S., an intemperate man, about fifty years of age, fell from a height, and broke both bones of the leg at the most common points of fracture in such cases—the fibula four inches, and the tibia two inches above the ankle-joint. The tibia penetrated the stocking and boot of the patient, and entered the soil, becoming denuded of periosteum to the extent of about one-fourth of an inch. One small detached fragment of the tibia was removed by the fingers; the wound closed by adhesive strips, scultetus bandage applied, one carved, anterior, and two long, lateral, well padded splints placed on the limb, the whole secured by strong tapes, the leg stretched on a pillow, ordered to be kept wet with spirits and water, and an anodyne administered. The patient rested tolerably well through the night, was free from pain in the morning, was removed to the Alms House under the superintendance of Dr. Shipman, and was left in bed by him, after an examination of the dressings. No large vessel or nerve was injured.

Nine days afterwards, Dr. S. was called to amputate, or assist in amputating the limb. A consultation of five surgeons, four students, and two non-medical superintendants! [what business had they at a medical consultation?] was called on the case.

*Condition of the limb.*—It lay on the double inclined plane, the bone protruding nearly two inches. It was dry, dark, and dead. The foot turned off—the leg distorded—muscles retracted—and the limb shortened. The wound gaped; there was no loss of substance; the upper part had healed, and healthy granulations covered a portion of the bone. [How so, if it "had lost its vitality?"] Some good pus escaped. The patient suffered much pain, but had no fever. The strength and pulse good—tongue clean—and bowels regular. Drs. Goodyear and Hyde, the attending surgeons, urged immediate amputation, on account of the habits of the patient, the state of the weather, and the fear of fever. One gentleman seemed in doubt. Three, including Dr. S., opposed amputation, and advised removing the protruding portion of bone, and reduction.

Ten days after the consultation, Dr. S. was called to take charge of the case—the attending surgeons being dismissed by the superintendents. *Condition of the patient* much the same. *The heel sloughed to the bone from pressure.* The limb was covered as before with a loose cloth. Much pain suffered.

The doctor removed about an inch of the protruding bone, including the part that had lost its vitality, placed the limb in an easy position and left. As another surgeon had been requested to assist in the treatment, *it appears that the limb was not regularly dressed till next day!* Then, the wound was "cleaned from pus and *maggots*," closed, as nearly as possible, with adhesive strips, and dressed, as was also the heel, and splints were applied. The patient then slowly recovered, leaving the Alms House *in the spring*, walking without much difficulty or much lameness.

This is a fair abstract of Dr. Shipman's own account of the case. Three letters follow—one, *very severe*, from Hon. Lewis Riggs, M. D., another from A. Patterson, M. D., and a third from Joel K. Carpenter, M. D. The first and last were of the consultation—the remaining gentleman had seen the case in its earlier progress, and participated in its later treatment. Besides a general corroboration of the statement of Dr. Shipman, these letters fully establish the following facts. The neglect of attention to the dressing of the wound; the absence of support to the foot, which seems to have been permitted to rotate the lower fragments almost at pleasure; and the uncommon capacity of endurance in the patient. A small sinus remained, but the patient was able to perform the duties of a labourer.

Nothing of much moment was elicited from other evidence on the trial, except that lateral splints of some kind were applied on the inclined plane, and that compresses in some shape were placed between these and the limb. There is much in the reported medical evidence on trial which it would have been desirable for some of the practitioners to suppress: but as the mode of reporting in our courts is such as has been described, charit and professional pride should induce us to attribute it to the ignorance of the unprofessional scribe. It is unnecessary to the formation of the few opinions which we feel it incumbent upon us to express.

If the statements of the operator and the three letter writers be correct, there can be no doubt that the condition of the patient on the 13th of July was such as to make it very proper that amputation should "*be talked of*," but we are acquainted with no experienced surgeon in this section of the country who would have ventured *to perform it* in the then existing state of the constitution. If, as some of the witnesses state, the wound looked "dark coloured," and "had the appearance of sloughing,"—even if it were *actually sloughing* to a moderate extent, while there were no worse constitutional symptoms, *we* should have excised the dead bone, reduced it, and expected with some confidence to see the necessity of wearing a high healed shoe, the worst ultimate consequence of the avoidance of amputation in a sturdy labourer from the country who had so long escaped mania a potu, and resisted so much local irritation without a fever of reaction or excessive suppuration. But, it must be admitted that a difference of opinion on this subject would not be just ground for a charge of ignorance or even severe censure. There are other more formidable questions. How came the bone to be protruding on the 13th, after being reduced perfectly, or even—for argument's sake—imperfectly reduced on the 4th? How came the limb to be permitted to remain in the same condition, or,—if placed in a proper position,—to resume its deformity in the interval between the 13th and 23d? Why was the foot so partially dressed? It is altogether unnecessary to reply to these queries.

It would be no more than just, however, to inquire of Dr. S—— why the dressing of the wound was delayed a day after the reduction on the 13th, in order to await the arrival of a distinguished surgeon, whose presence could be of no avail in the execution of a task so simple.

Disliking as we do decidedly, the plan of treating fractured legs by means of short splints, and especially carved splints, and condemning the double inclined plane still more decidedly, we cannot avoid thinking that had the wound been simply dressed with adhesive strips, the leg reduced and early placed on a pillow in the common fracture box, and covered with bran, without any bandage, the patient would have left the alms-house in three or four months with a full length limb, and, at most, a little sinus communicating with an insignificant sequestrum. There would been, in this case, much less trouble in dressing, and no danger of maggots. Not above one case in twenty of fractured leg requires permanent extension, and in the Pennsylvania Hospital no instrument but the fracture box is employed in cases in which the length of the limb is spontaneously preserved. If the tibia had been denuded in the first instance to the extent of an inch, this circumstance alone would not have warranted the excision of the bone, unless the medullary membrane were seriously torn, as the consequent exfoliations would have been superficial. But some of these latter views are in contradiction to very high authority, and no charge of mal-practice could be maintained upon the strength of their rejection by the practitioner.

